# Genome Sequence of *Flavobacteriaceae* Strain W22, Isolated from a Tree Hole Mosquito Habitat

**DOI:** 10.1128/MRA.00008-20

**Published:** 2020-02-13

**Authors:** Shicheng Chen, Edward D. Walker

**Affiliations:** a Department of Microbiology and Molecular Genetics, Michigan State University, East Lansing, Michigan, USA; b Department of Entomology, Michigan State University, East Lansing, Michigan, USA; Broad Institute

## Abstract

The bacterium *Flavobacteriaceae* strain W22 was isolated from the water column of a tree hole larval habitat of the mosquito Aedes triseriatus. The draft genome contained 3,796,379 bp, a G+C content of 35.4%, and several genes encoding enzymes functioning in polysaccharide degradation, gliding motility, and the type IX protein secretion system (T9SS).

## ANNOUNCEMENT

*Flavobacteriaceae*, the largest family in the phylum *Bacteroidetes*, contains up to 90 genera and hundreds of species (1). One of the important features of members of *Flavobacteriaceae* is that they catabolize various biomacromolecules, including polysaccharides and proteins (2). *Flavobacteriaceae* strain W22 was isolated from the water column of a tree hole in the root buttress of an American beech tree. Such tree holes form the native habitat of larvae of the mosquito species Aedes triseriatus in eastern North America.

Strain W22 was cultured in Casitone yeast extract (CYE) medium under aerobic conditions at 22°C, followed by genomic DNA extraction using the Wizard genomic DNA purification kit (Promega, CA, USA) (3). Next-generation sequencing (NGS) libraries were constructed using the Illumina TruSeq nano DNA library preparation kit, following standard procedures recommended by the manufacturer. The draft genome of *Flavobacteriaceae* strain W22 was sequenced by Illumina MiSeq paired-end sequencing technology at the Research Technology Support Facility (RTSF) at Michigan State University. The sequencing was performed in a 2 × 250-bp paired-end format using a v2, 500-cycle reagent cartridge. The raw sequences with 464,682,000 bp were trimmed using Trimmomatic (version 0.38; settings, paired-end mode with a window size of 4, quality requirement of 15, and minimum read length of 36) (4). The trimmed sequences were further reanalyzed using FastQC version 0.11.9, which led to 201 contigs with an *N*_50_ value of 115,558 bp. The reads were assembled using SPAdes (version 3.13) (5) with default settings. After removing short sequences (less than 500 bp), it resulted in 65 contigs. The assembled genome comprised 3,796,379-bp nucleotides with a G+C content of 35.4% (>50-fold coverage). Gene annotation was carried out by the NCBI Prokaryotic Genome Automatic Annotation Pipeline (PGAAP; version 4.10) (https://www.ncbi.nlm.nih.gov/genome/annotation_prok/). There were at least 56 RNA sequences, including 50 tRNAs, 3 rRNAs, and 3 noncoding RNAs (ncRNAs), in the genome.

According to the 16S rRNA nucleotide sequence analysis (extracted from assembled genome), W22 is most closely related to species of the genus *Chryseobacterium* (87.5% to 90.2% similarity) within the family *Flavobacteriaceae*. W22 genome sequences were used to search the NCBI database by genome BLAST. Sequences producing significant alignments included only those from *Chryseobacterium* spp. The W22 genome showed average nucleotide identities of 91.58%, 80.85%, and 80.62% to those of Chryseobacterium gleum strain 3012STDY6944375 (GenBank accession number NZ_LR215974.1), Chryseobacterium balustinum strain KC_1863 (NZ_CP033934.1), and Chryseobacterium indoltheticum strain ATCC 27950 (NZ_CP033929.1), respectively. Therefore, W22 very likely does not belong to the genus *Chryseobacterium* (16S rRNA similarity, <91%; average nucleotide identity [ANI], <92%), and it may properly be assigned to a different genus, as yet undetermined.

*Flavobacteriaceae* strain W22 had many genes encoding proteins involved in gliding motility and the type IX protein secretion system (T9SS) (6). It carried several genes encoding enzymes that catabolize certain polysaccharides (endo-1,4-β-xylanase, α-amylase, neopullulanase, β-glucosidase, and 1,4-α-glucosidase) (2). Furthermore, large genetic elements of conjugative transposons, including *TraA*, *TraB*, *TraC*, *TraD*, *TraE*, *TraF*, *TraG*, *TraI*, *TraJ*, *TraK*, *TraL*, *TraM*, *TraO*, and *TraQ*, were present (7).

### Data availability.

The genome sequence of *Flavobacteriaceae* bacterium W22 was deposited in GenBank under accession number WUWH00000000. The BioProject designation for this project is accession number PRJNA598108. The BioSample accession number is SAMN13698508. The raw sequence data are available under SRA accession number SRR10874259.
